# Chitosan-Nanoencapsulated Curcumin for the Treatment of Diabetic Foot Ulcers: A Review

**DOI:** 10.3390/polym18040511

**Published:** 2026-02-19

**Authors:** Laura Andrea Gómez-de la Cruz, Juan David Rodríguez Macías, Carlos David Grande-Tovar

**Affiliations:** 1 Grupo de Investigación de Fotoquímica y Fotobiología, Programa de Química, Universidad del Atlántico, Carrera 30 No. 8–49, Puerto Colombia 081007, Colombia; lauragomezdlc@hotmail.com; 2 Programa de Medicina, Facultad de Ciencias de la Salud, Exactas y Naturales, Universidad Libre, Km 5 Vía Puerto Colombia, Barranquilla 081007, Colombia; juand.rodriguezm@unilibre.edu.co

**Keywords:** curcumin, chitosan, diabetes, diabetic foot ulcer, nanoencapsulation

## Abstract

Diabetic foot ulcers (DFUs) are wounds characterized by chronic inflammation and elevated oxidative stress that delay tissue regeneration and render them susceptible to infection, thereby complicating healing. Therefore, treating DFUs effectively is often challenging and requires a combined approach that integrates anti-inflammatory, antioxidant, and antibacterial effects. Curcumin, a widely studied natural compound, has shown promise in wound healing by modulating inflammation, oxidative stress, and infections. However, its bioavailability, absorption, and solubility issues limit its clinical applications. To overcome these limitations, curcumin has been incorporated into nanosystems, such as hydrogels, nanofibers, nanoparticles, vesicles, and micelles, thereby improving its delivery and enabling efficient local administration. Among these nanosystems, those formulated with chitosan are of particular interest due to chitosan’s intrinsic wound-healing properties. For that reason, this review comprehensively analyzes the literature on the therapeutic mechanisms of the chitosan–curcumin system for diabetic wound closure and compares them with those of free curcumin. The results show that this system exerts anti-inflammatory, antioxidant, and antimicrobial effects through specific mechanisms, including macrophage polarization, modulation of oxidative stress, and alteration of bacterial cell walls. In addition, significant improvements are observed in key healing processes, including cell migration, fibroblast proliferation, collagen deposition, and re-epithelialization. It should be noted that chitosan not only promotes curcumin release but also contributes to its therapeutic effect through its inherent antimicrobial and hemostatic properties, reinforcing its potential as a comprehensive strategy for the treatment of DFUs.

## 1. Introduction

Diabetes is a common chronic metabolic disease. According to the International Diabetes Federation (IDF), 590 million people worldwide have been diagnosed with diabetes, a figure that is projected to rise to 853 million by 2050 [1].

A serious complication of diabetes is diabetic foot ulcer (DFU), a type of wound associated with high levels of reactive oxygen species (ROS), an imbalance between pro-inflammatory and anti-inflammatory cells and factors, and poor angiogenesis [2].

Compared with normal skin wound healing, diabetic wounds tend to heal more slowly, making them more susceptible to infection [2]. Infections of diabetic foot ulcers are the most common complication, as in most cases they require hospitalization and can be so severe that they necessitate limb amputation [3]. Effective treatment of DFUs is complex and costly for healthcare systems in countries. DFUs are usually treated using a multimodal strategy that includes glycemic control, topical and systemic antibiotics, wound debridement, and promotion of ulcer healing through dressings [4]. Given that diabetic wounds are multifactorial, interventions aimed at treating a single symptom may be insufficient to achieve wound healing. In this regard, the most effective approach to promoting diabetic wound healing is to develop therapeutic strategies that combine anti-inflammatory, antioxidant, and antibacterial effects [2].

Curcumin is a natural polyphenolic compound found in high proportions (77%) in the rhizomes of *Curcuma longa* and *Curcuma aromatica* [2]. This substance has been extensively studied in applications such as anticancer [5], antidiabetic [6], hepatoprotective [7,8], anti-aging [9], among others, standing out for its potential as a wound healing agent [10], as it protects the wound from bacterial infection, reduces inflammation, and increases cell proliferation, thereby promoting the reconstruction of damaged tissue. Curcumin also has antioxidant activity, which enhances its effects as a healing agent, since free radicals promote wound inflammation and delay the natural healing process [11]. Thus, curcumin accelerates wound healing by contributing to the inflammatory, proliferative, and remodeling phases of wound healing, and, together with its anti-infective, anti-inflammatory, and antioxidant properties, makes it a promising treatment for complex wounds, such as diabetic foot ulcers. Recent studies have shown that curcumin can reduce inflammation and promote wound healing in diabetic mice, even in infected wounds [12].

Despite the proven effects of curcumin in tissue regeneration applications, its low bioavailability, limited absorption, poor solubility, and toxicity at high concentrations in topical applications limit its clinical use [13,14]. To overcome these limitations, several studies have proposed the formulation of curcumin with various nanoscale materials to improve its bioavailability, enable sustained administration at therapeutic levels, and facilitate local administration [15]. Among these nanosystems, polymeric nanoparticles [16], mesoporous nanoparticles [17], nanoemulsions [18], and liposomes stand out [19].

Among these materials, chitosan is of particular interest because, in addition to being biocompatible, non-toxic, and bioresorbable, it has biological activity, notably antimicrobial activity [20]. Furthermore, the composition of this biopolymer, composed of monomeric units of N-acetylglucosamine, promotes hemostasis, stimulates cell proliferation, and, as a result, accelerates wound healing [21]. However, to optimize its properties for tissue regeneration applications, it must be combined with bioactive substances that provide anti-inflammatory and antioxidant activity, such as curcumin [22].

The nanoencapsulation of curcumin with chitosan (Cur-CS-NP) has been extensively studied, with promising findings regarding its use for the healing of chronic wounds in diabetic patients. A significant decrease in macrophage-mediated inflammation at the wound site has been reported [11]; in addition, the impact of these nanoparticles on the generation of reactive oxygen species (ROS), cytokine production, and inflammatory biomarker expression in mouse T cells or human macrophages has been explored, showing decreased production [23]. These studies demonstrate that the nanoencapsulation of curcumin with chitosan optimizes its anti-inflammatory, antimicrobial, and antioxidant properties. However, little is known about the mechanisms by which nanoencapsulated curcumin reduces inflammation and promotes wound healing, as well as its long-term effects. Therefore, this review examines the literature on mechanisms underlying improved healing, compares Cur-CS-NPs with free curcumin for tissue regeneration in DFU patients, and outlines their advantages, providing high-value information for physicians, scientists, and patients worldwide.

## 2. Materials and Methods

### 2.1. Databases

This study compiled information from three databases: Scopus, Web of Science (WoS), and Google Scholar. The bibliographic search in Scopus was performed using the following search key: TITLE-ABS-KEY ((curcumin AND (nano* OR “nanoparticle*” OR “nanomaterial*”) AND (antioxidant* OR inflammat* OR antimicrobial * OR “tissue regeneration” OR “tissue engineering” OR “diabetic wound”))) AND (“chitosan”). For the WoS and Google Scholar databases, the search key included the terms: “chitosan,” “curcumin,” “nano,” “nanoparticle,” “wound,” “regeneration,” “diabetic,” “antioxidant,” “anti-inflammatory,” and “antimicrobial”.

This search was refined by limiting the results to documents published since 2019 that did not belong to the categories of review, meta-analysis, or systematic review, and by focusing specifically on the category of “articles” in the areas of interest: “Medicine Research Experimental,” “Pharmacology Pharmacy,” “Biotechnology Applied Microbiology,” “Nanoscience Nanotechnology,” “Biology,” and “Microbiology.” A total of 266 documents were extracted up to February 2025. The data were processed using Mendeley Reference Manager to remove duplicates.

On the other hand, we used VOSviewer (www.vosviewer.com; Van Eck and Waltman, 2009–2022, version 1.6.20, Leiden University, Leiden, The Netherlands), which is open-source software, to create network maps of institutions, countries, keywords, and citations per article [24]. We accessed VOSviewer on 3 February 2026. Network maps were created for the keywords “curcumin delivery systems: wound healing” and “curcumin delivery systems with chitosan: wound healing,” covering the period from 2020 to date.

### 2.2. Study Selection

Articles in English that included in vivo and in vitro studies were considered for the review. These articles had to address the antioxidant, anti-inflammatory, or antimicrobial properties of curcumin and chitosan, individually or in combination. Articles investigating the healing activity of nanostructures formed by curcumin and chitosan, which may or may not be associated with other components, in the healing of common wounds and DFU were also included.

Studies with information on nanostructures used in non-therapeutic fields and in applications other than wound healing or tissue regeneration were excluded, as were articles in which other active ingredients accompanied curcumin. For the first inclusion criterion, 184 documents that did not focus on wound healing were excluded; in the second information filter, nine records that did not have therapeutic purposes were discarded; on the other hand, studies with active ingredients other than curcumin (11 records) were excluded, resulting in a total of 60 articles, which were used to construct this review (Scheme S1, Supplementary Materials).

Finally, to conduct a detailed analysis of the most relevant information on the mechanisms underlying the antioxidant, anti-inflammatory, and antibacterial properties of chitosan–curcumin nanostructures in the context of DFU healing, a comprehensive review of the results was carried out. To this end, the data were analyzed and presented in specialized sessions. These sessions addressed issues ranging from DFU healing to the development and application of these systems in the treatment of DFUs.

## 3. Problems with Healing Ulcers in Diabetic Feet

**Figure 1:**
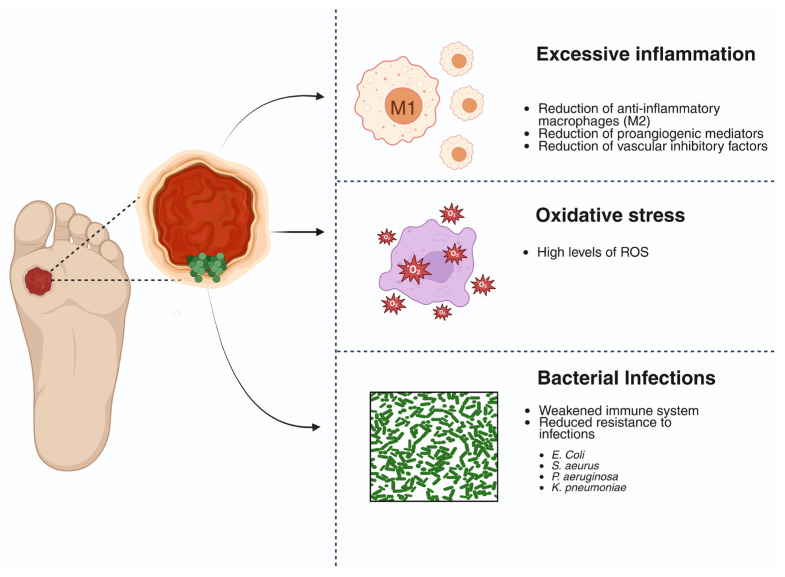
The main factors delaying healing of diabetic foot ulcers (DFU). Created in BioRender. Grande tovar, C. D. (2026) https://BioRender.com/l57tv5g, 16 January 2026.

The healing of diabetic foot ulcers can be delayed by various hyperglycemia-related factors, including high levels of reactive oxygen species (ROS), excessive inflammation, sustained hypoxia, and bacterial infections (Figure 1) [25,26]. This pathophysiological environment can cause an acute wound to become chronic [27].

ROS can promote epithelial cell migration and improve healing; however, excess ROS produce superoxide anions that damage tissue and inhibit the activity of antioxidant enzymes, causing oxidative stress, prolonging the inflammatory phase, and impairing angiogenesis [28]. Chronic hyperglycemia increases ROS production through metabolic pathways, such as the polyol pathway and protein kinase C (PKC)-mediated signaling, which, in turn, leads to redox overload that exceeds endogenous antioxidant capacity [29]. This excessive and uncontrolled oxidative stress promotes the oxidation of lipids, proteins, and DNA, thereby affecting cellular integrity and prolonging chronic oxidative stress, which ultimately impairs the normal function of endothelial cells and fibroblasts, which are necessary for tissue regeneration [30].

Oxidative stress also generates a persistent inflammatory microenvironment characterized by an excess of inflammatory macrophages (M1) and a reduction in anti-inflammatory macrophages (M2). Sustained polarization toward the M1 phenotype causes an M1/M2 imbalance that prevents wounds from transitioning to the proliferative phase and compromises the formation of functional granulation tissue [31]. This imbalance is accompanied by decreased levels of proangiogenic mediators, including transforming growth factor-α (TGF-α), TGF-β, fibroblast growth factor-2 (FGF-2), vascular endothelial growth factor (VEGF), epidermal growth factor (EGF), hypoxia-inducible factor-1α (HIF-1α), and vascular inhibitory factors. Overall, these changes lead to inadequate angiogenesis and delayed wound healing [32,33,34].

The combination of oxidative stress and chronic inflammation creates an unfavorable environment for tissue repair, angiogenesis, and cell migration, thereby keeping wounds open and exposed to infection [35]. Bacterial infections significantly alter healing and are very common among diabetic patients, whose chronic hyperglycemia affects the immune system, reducing resistance to infections and promoting bacterial growth, further complicating healing [2,36]. In chronic ulcers, these infections are usually polymicrobial, with *Staphylococcus aureus*, *Escherichia coli*, *Pseudomonas aeruginosa*, and *Klebsiella pneumoniae* among the most common pathogens [37]. These bacteria stimulate a sustained inflammatory response and ROS production, perpetuating the cycle of healing inhibition [38]. In addition, they can form biofilms that promote inflammation and inhibit cell migration, hindering the wound’s progression toward the resolution phases [39].

## 4. Anti-Inflammatory, Antioxidant, and Antibacterial Properties of Curcumin

### 4.1. Curcumin as an Anti-Inflammatory

**Figure 2:**
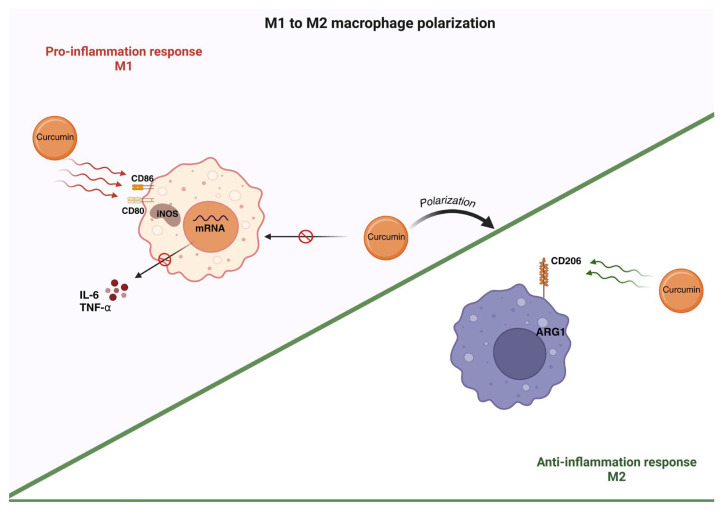
Immunomodulatory role of curcumin in macrophage polarization during DFU. Abbreviations: CD86, cluster of differentiation 86; CD80, cluster of differentiation 80; CD206, cluster of differentiation 206; iNOS, inducible nitric oxide synthase; IL-6, interleukin-6; TNF-α, tumor necrosis factor alpha; ARG1, arginase 1; mRNA, messenger RNA. Curved green arrows denote increased activity, curved red arrows denote decreased activity, and blocking arrows denote inhibition. Created in BioRender. Grande tovar, C. D. (2026) https://BioRender.com/m9slb0n, 16 January 2026.

Curcumin has been the subject of numerous studies evaluating its therapeutic properties, especially its anti-inflammatory activity [40,41,42]. One of the most notable mechanisms is the modulation of macrophage polarization. Curcumin has been shown to induce autophagy, promoting the transition from proinflammatory M1 macrophages to anti-inflammatory M2 macrophages. It has also been shown to affect the expression of surface markers (increased ARG-1 and CD206, decreased iNOS, CD80, and CD86), which are key to modulating macrophages towards the M2 phenotype, leading to the resolution of inflammation [43]. This mechanism is illustrated in Figure 2.

**Figure 3:**
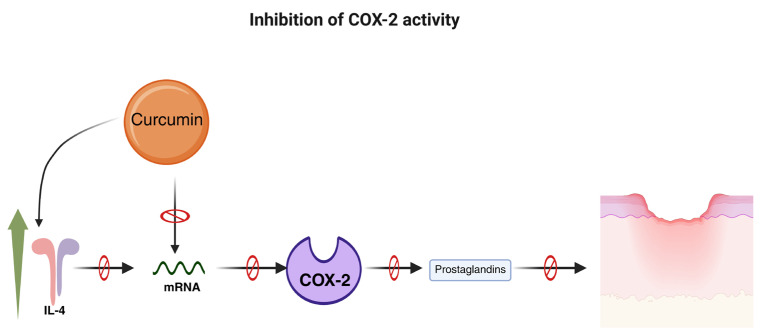
COX-2 inhibition by curcumin as a mechanism for inflammation reduction in DFU. Abbreviations: COX-2, Cyclooxygenase-2; IL-4, interleukin-4; mRNA, messenger RNA. Blocking arrows indicate inhibition. The green arrow indicates upregulation of IL-4. Created in BioRender. Grande tovar, C. D. (2026) https://BioRender.com/jxdfuzr, 16 January 2026.

On the other hand, inhibiting key enzymes in the inflammatory cascade is also an important mechanism. It is well known that the anti-inflammatory effects of curcumin are mainly mediated by selective inhibition of the mRNA expression of the enzyme cyclooxygenase-2 (COX-2) [44,45]. Blocking this enzyme’s activity limits the synthesis of prostaglandins and other inflammatory mediators derived from arachidonic acid, resulting in significant improvements in inflammation [46]. The high affinity of curcumin for this enzyme has been demonstrated through in silico studies and is associated with reduced inflammation (*p* ≤ 0.05) in rats supplemented with curcumin [47]. The suppression of this enzyme could also be related to curcumin’s ability to significantly increase anti-inflammatory cytokines, such as interleukin-4, which correlates directly with COX-2 inhibition and COX-2 mRNA [48]. Figure 3 provides a schematic description of this curcumin’s antioxidant mechanism.

**Figure 4:**
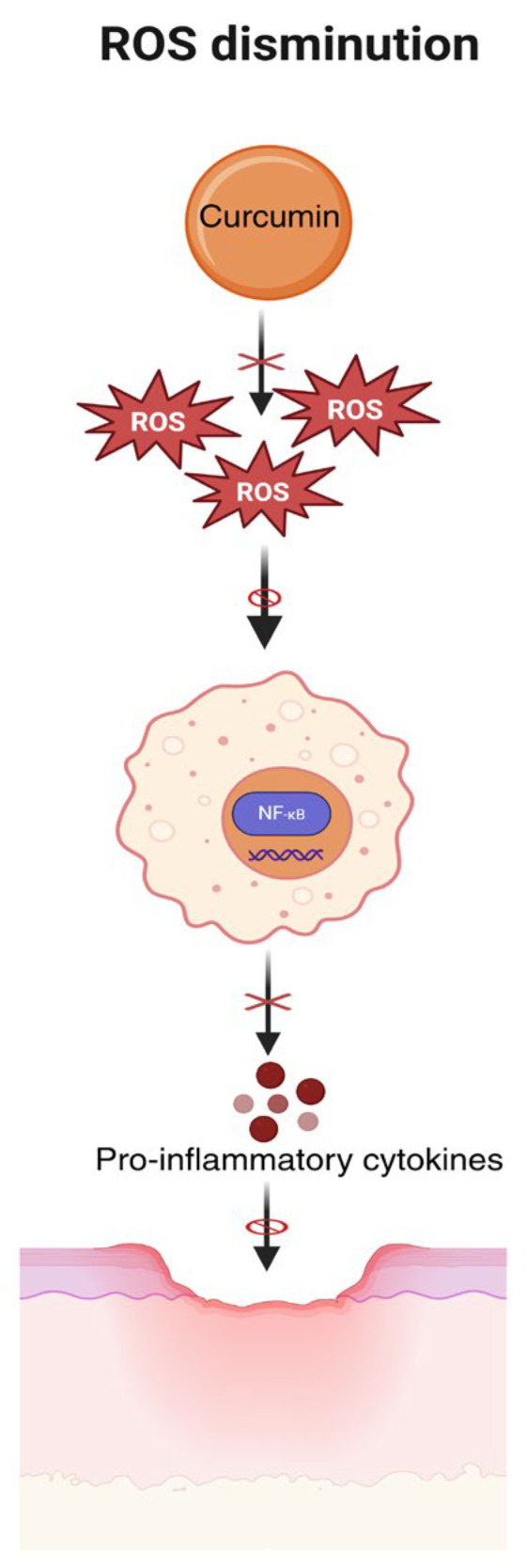
Reduction in oxidative stress by curcumin through ROS/NF-κB signaling in UPD. Abbreviations:. NF-Kb, Nuclear factor κB. Created in BioRender. Grande tovar, C. D. (2026) https://BioRender.com/elcxw87, 16 January 2026.

Likewise, a decrease in ROS leads to negative regulation of nuclear factor kappa B (NF-κB), a factor known to trigger the production of inflammatory cytokines, improving inflammation [33]. A visual summary of this mechanism is shown in Figure 4.

In turn, mechanisms such as inhibition of protein denaturation and decrease in the production of proinflammatory cytokines such as tumor necrosis factor alpha (TNF-α) and interleukin-6 (IL-6) at the messenger RNA level in macrophages have also been shown to be an essential mechanism of curcumin in mitigating the inflammatory response [49,50].

### 4.2. Curcumin as an Antioxidant

**Figure 5:**
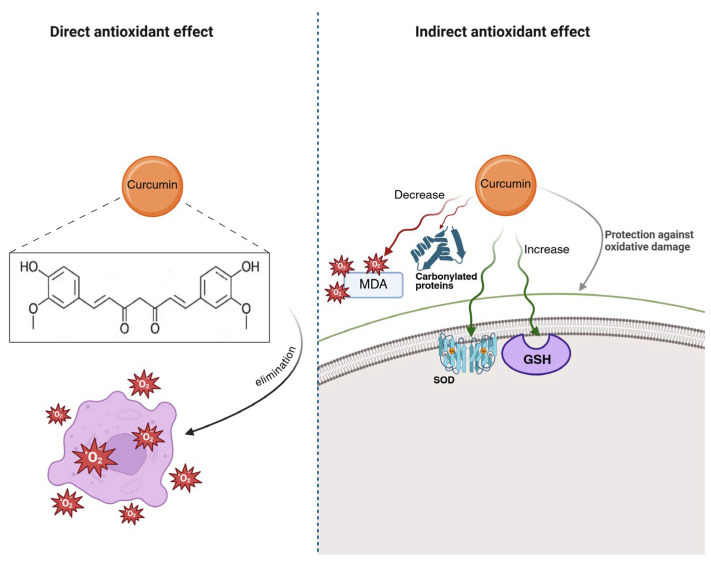
Antioxidant mechanisms of curcumin regulating oxidative damage in DFU. Abbreviations: MDA, malondialdehyde; GSH, glutathione peroxidase; SOD, superoxide dismutase. Created in BioRender. Grande tovar, C. D. (2026) https://BioRender.com/lh2xwd0, 16 January 2026.

The antioxidant properties of curcumin have been extensively studied. It has been shown that the presence of hydroxyl and phenolic groups in its structure gives the molecule the ability to act as an antioxidant, both directly by eliminating free radicals and indirectly by increasing the activity of key enzymes [51] (Figure 5). Indirectly, curcumin can increase the activity of enzymes such as superoxide dismutase (SOD) and glutathione peroxidase (GSH) [52,53]. This action is evidenced by decreases in serum levels of key markers, such as thiobarbituric acid-reactive substances (TBAR) and the enzyme GSH [46]. In addition, it protects against oxidative damage by reducing malondialdehyde (MDA) and carbonyl protein levels, suppressing lipid peroxidation, and increasing total antioxidant capacity [51,52].

The antioxidant properties of curcumin also exert direct effects on cell viability under oxidative stress. A recent study reported that curcumin reduces mitochondrial damage, lipid peroxidation, and apoptosis in HS68 fibroblasts exposed to hydrogen peroxide (H_2_O_2_). These fibroblasts exhibited a significant decrease in viability (53%) when exposed to the oxidizing medium; however, when cultured in curcumin extracts, viability increased to up to 80%, indicating significant protection against oxidative damage [33].

It has also been shown to induce antioxidant defense mechanisms through gene regulation, including Nrf2 transcription factor activation, which promotes the expression of enzymes such as paraoxonase 1 (PON1), heme oxygenase 1 (HO1), and glutathione [52,54].

### 4.3. Curcumin as an Antibacterial Agent

Currently, antibiotic resistance has progressively reduced the therapeutic efficacy of antibiotics, creating a serious public health problem, especially in the context of infected local wounds, which are often treated with systemic antibiotics. This problem has prompted the search for alternative antibiotics, primarily for local use.

In this search, curcumin has been shown to have promising antimicrobial effects against a wide range of pathogens, including bacteria such as *Staphylococcus aureus*, *Staphylococcus haemolyticus*, *Escherichia coli*, *Proteus mirabilis*, *Streptococcus pyogenes*, *Acinetobacter lwoffii*, *Pseudomonas aeruginosa*, and *Enterococcus faecalis* [55], *Bacillus subtilis*, *Salmonella thyphimurium*, *Serritia mercescens*, *Proteus vulgaris* [56], *Trichophyton gypseum*, *Salmonella paratyphi*, and *Mycobacterium tuberculosis* [57]. Among these, *S. aureus* and *E. coli* have been the most studied and exhibit the most significant inhibition by curcumin [58].

**Figure 6:**
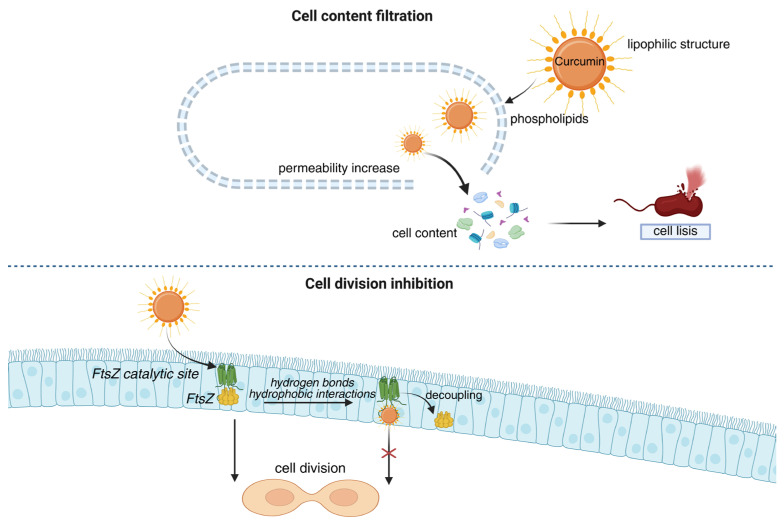
Antibacterial mechanisms of curcumin relevant to infection control in DFU. Abbreviations: FtsZ, temperature-sensitive filamentous mutant Z. The arrow marked with an “X” indicates inhibition of bacterial cell division. Created in BioRender. Grande tovar, C. D. (2026) https://BioRender.com/0q94zzl, 16 January 2026.

Although inhibitory effects have been observed in both gram-positive and gram-negative bacteria, curcumin tends to be more effective against gram-positive bacteria due to differences in their cell wall structures [59]. Curcumin exerts its antimicrobial activity through multiple mechanisms, as illustrated in Figure 6.

The primary mechanism is curcumin’s lipophilicity, which allows it to insert into the lipid bilayers of bacterial cell membranes, altering their structure and disrupting biochemical processes that increase permeability. This permeabilization allows the cellular contents to be filtered, ultimately causing cell lysis [43,55,60].

Curcumin has also been shown to exert its bactericidal action by interacting with the temperature-sensitive filamentous mutant (FtsZ), a key protein in cell division [55]. Its phenolic group, as well as the methoxy and carbonyl functional groups, interact with the catalytic site of FtsZ via hydrogen bonds and nonspecific hydrophobic interactions (Figure 6), thereby inhibiting cell division [56].

The interaction between curcumin and the central catalytic domain of FtsZ was investigated in *E. coli* and *B. subtilis* strains using computational docking, molecular electrostatic potential (MEP) analysis, and cavity depth (CD) studies [61]. The results indicated that curcumin binds to the active site of FtsZ in both strains through interactions with the catalytic residues glycine, threonine, and asparagine. The symmetrical structure of curcumin facilitates this binding. In particular, the terminal phenolic moiety and keto oxygens form hydrogen bonds with the catalytic residues [61]. Other mechanisms include inhibition of bacterial DNA replication and modification of gene expression [54,55]. All these effects are dose-dependent, as reported [60].

In the field of nanoformulation, it has been shown that the encapsulation system affects curcumin’s selectivity against specific strains [62], as well as its minimum inhibitory concentration (MIC) [63] and effectiveness [13]. Interactions with bacterial cell membranes vary across systems, suggesting changes in efficacy and selectivity.

In acidic microenvironments, such as those found in bacterial infections, certain nanocarriers release curcumin in a sustained manner, thereby maximizing its bactericidal activity [44,57]. The development of nanocarriers sensitive to exogenous or endogenous stimuli (e.g., temperature, pH, ROS levels, ultrasound, magnetic fields, and light), particularly pH, is relevant to the topical and localized treatment of infections [13,62]. These effects are exciting in applications such as photodynamic therapy, where curcumin acts as a photosensitizer, capable of converting molecular oxygen into singlet oxygen under specific light irradiation. This reactive species is highly toxic to bacteria, inactivating and killing them. This therapy helps treat isolated infections in wounds and improve wound healing [64,65].

## 5. Curcumin Delivery Systems for Tissue Regeneration in DFUs

A bibliometric analysis was conducted to identify research trends and thematic relationships in the literature on curcumin delivery systems for wound healing. This analysis was based on a keyword co-occurrence network, which enabled visualization of dominant research topics and their interconnections.

**Figure 7:**
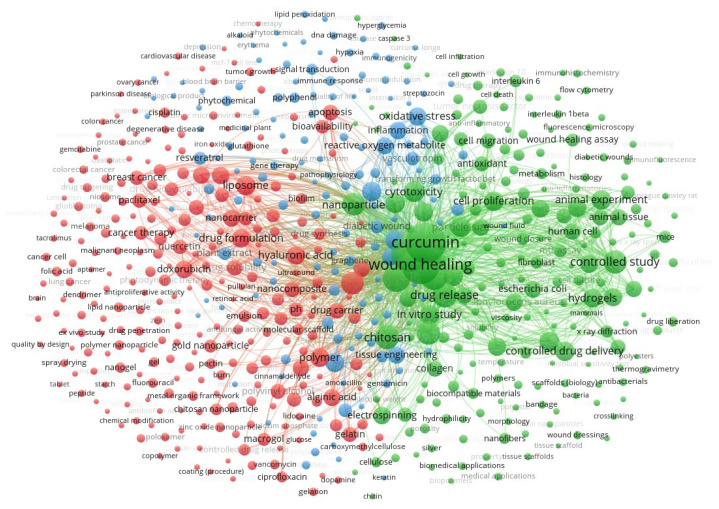
Co-occurrence network based on article weights for terms associated with the first group. The curved lines of varying thickness, representing co-occurrence, illustrate the relationships between the terms. The proximity between the elements reflects the strength of their connection, while the size of each term is determined by its frequency of occurrence: Keywords: 615. Clusters: 3. Links: 124,704. Total link strength: 515,502.

The keyword network was constructed from terms cited at least 10 times, comprising 615 words organized into three groups, with a total link strength of 515,502 (Figure 7). This set of words was obtained by removing terms such as “cardiovascular diseases” and “osteoarthritis,” which correspond to pathologies outside the scope of this review. The first cluster, represented in green, identifies terms related to the therapeutic properties of curcumin and its biological implications. The second cluster, in red, concerns drug delivery systems and exhibits diverse research without discernible major clusters. The blue cluster has an even lower density and primarily addresses curcumin’s role in regulating reactive species.

Keyword analysis provides information on the topic’s relevance to current research. In this context, there is a clear trend across the clusters toward curcumin’s therapeutic properties. This finding reflects the growing interest in strategies to overcome curcumin’s limited bioavailability and maximize its biological effects.

**Figure 8:**
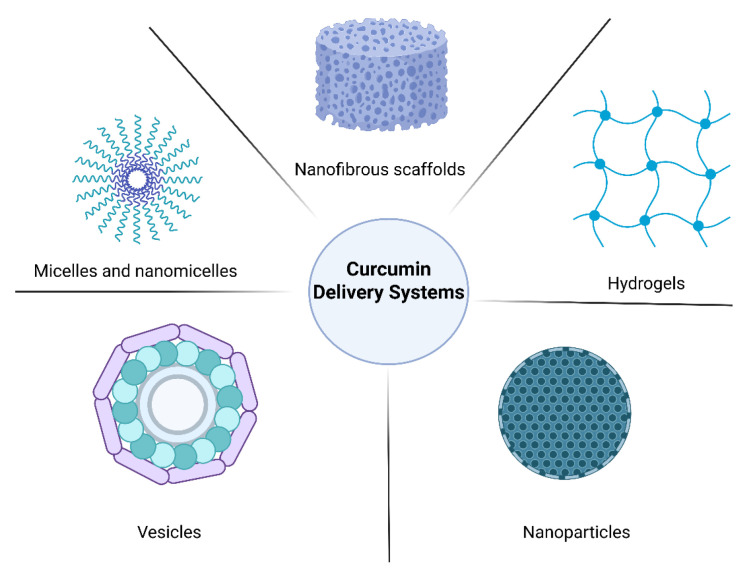
Curcumin delivery systems for tissue regeneration in DFU. Created in BioRender. Grande tovar, C. D. (2026) https://BioRender.com/8511db3, 16 January 2026.

Consequently, as illustrated in Figure 8, various delivery systems, such as hydrogels, emulsions, nanoparticles, and micelles, have been reported in the literature for delivering active ingredients with bioavailability issues, and can also provide functional characteristics such as sustained release, biocompatibility, and biofunctionality [66].

### 5.1. Hydrogels

Hydrogels are hydrophilic polymeric systems that can absorb and retain large amounts of water within their internal structure without losing their integrity [67]. Interest in these systems in tissue regeneration is mainly due to their ability to mimic the extracellular matrix (ECM), provide a favorable biochemical environment, and offer mechanical support to tissues [68]. In particular, hydrogels offer several advantages for wound healing, including high retention capacity, good swellability, controlled drug release, biocompatibility, and adhesive properties, which enable them to adapt to skin movement and act as physical barriers to prevent bacterial invasion. In addition, they maintain a moist environment in the wound, which accelerates the overall healing process [66,69].

The successful development of these systems with curcumin involves combining different biomaterials, the most used of which are chitosan, cellulose, hyaluronic acid, fibrin, collagen, and gelatin [70]. Several of these hydrogels have been reported in the literature [71]. For example, a hydrogel scaffold composed of carboxymethyl-diethyl aminoethyl cellulose (CM-DEAEC) loaded with curcumin was reported as a wound dressing, exhibiting more than 99% antibacterial activity and high active ingredient loading efficiency (83%); in addition, it achieved sustained release and zero toxicity in fibroblast-like cells derived from L-929 mouse subcutaneous connective tissue [66].

In another study, curcumin nanoparticles were encapsulated in a chitosan–hyaluronic acid hydrogel, along with a bioactive peptide derived from the skin secretions of *Rana limnocharis*, called RL-QN15. This system demonstrated improvements in antibacterial activity, restructured the wound immune microenvironment, reduced inflammation, and promoted angiogenesis, resulting in accelerated healing of diabetic wounds within 17 days [69]. The importance of fostering a favorable immune microenvironment in the wound was also highlighted in the development of a thermosensitive methylcellulose hydrogel with carboxymethyl chitosan that integrated curcumin into the zeolite imidazolate framework-8 (ZIF-8). This pH-sensitive system continuously releases curcumin into the acidic wound environment, demonstrating strong resistance to bacterial infections, ROS elimination, and reduced inflammatory symptoms. In addition, this system underwent sol–gel phase transitions at human body temperature (in situ synthesis), which enables it to form an effective physical barrier for uniform filling of irregular wounds. These characteristics achieved a healing rate of over 97% on day 14 of treatment; therefore, this system shows potential for diabetic wound healing [72]. The antimicrobial properties were also evident in the development of a dressing composed of an acellular porcine dermal matrix (PADM) and a magnesium-loaded curcumin (PADM@MgC). The integrity of bacterial membranes was analyzed using scanning electron microscopy (SEM) and fluorescence microscopy, and it was observed that sustained curcumin release, induced by the wound’s acidic microenvironment, led to leakage of intracellular contents and significant membrane damage [43].

On the other hand, the design of a modified gelatin hydrogel dressing with gelatin nanoparticles coated with curcumin was reported, capable of providing an intelligent response under acidic conditions, especially in infected wounds, releasing curcumin in a sustained manner and significantly improving the antimicrobial properties, closure, healing, and vascularization of infected wounds in an animal model [73].

A reduction in oxidative stress was also demonstrated in a hydrogel system comprising a curcuminoid nanoemulsion and marine collagen peptides (CMPs). This gel exhibited potential antibacterial activity against MRSA (methicillin-resistant *Staphylococcus aureus*). It inhibited inflammation by promoting re-epithelialization during the healing of diabetic wounds infected with MRSA, especially at a curcuminoid-to-CMP ratio of 2:1 [74]. Finally, sprayable hydrogels were designed with hollow, porous ZnO microspheres and curcumin nanoparticles; these nanoparticles, dispersed in water, facilitated fibroblast migration and angiogenesis, making it a viable option for medical dressings [75].

### 5.2. Nanofibers

Nanofibers are continuous fibers with diameters in the nanometer range that form porous scaffolds resembling the extracellular matrix [76,77]. These structures have numerous advantages for the efficient delivery of active ingredients, including a higher surface-to-volume ratio, efficient encapsulation, reduced microbial contamination by minimizing aqueous activity, sustained release, and the ability to adsorb wound exudates [78]. In wound-healing applications, curcumin nanofibers are commonly formulated with materials such as poly (L-lactic acid) (PLLA), poly(caprolactone) (PCL), poly(lactide-co-glycolide) (PLGA), chitosan, and polyvinyl alcohol (PVA) using various techniques, primarily electrospinning. This technique is commonly used for its simplicity, low cost, high efficiency, and ease of scalability; it also provides control over morphology [77].

Nanofibers that include PLLA have been widely used [70]. A recent study showed that incorporating curcumin and silver nanoparticles into PLLA nanofibers promoted diabetic wound healing in vivo and improved their antioxidant, antibacterial, and anti-inflammatory properties in vitro by efficiently removing ROS and exerting immunomodulatory effects that protect cells from oxidative damage and reduce inflammation [2]. Likewise, a micro/nanofibrous scaffold loaded with curcumin and zinc ion eutectic metal–organic frameworks (MOFs) has been reported via electrospinning and crystal engineering for diabetic wound healing. These scaffolds release curcumin on demand in the early stage of wound healing; in addition, they possess excellent anti-inflammatory and antioxidant properties and promote angiogenesis and collagen deposition in vitro and in vivo [79].

In another study, PCL nanofibers were electrospun as a curcumin delivery vehicle. These fibers achieved sustained release for 72 h and a decrease in dose below the reported cytotoxic concentration; they also maintained the viability of HFF-1 cells (human foreskin fibroblasts-1) above 70% under oxidative stress conditions, reduced inflammation, and demonstrated a higher wound closure rate in a model of streptozotocin-induced diabetic mice [15]. Likewise, the development of a multifunctional electrospun nanofiber composed of chitosan and polyvinyl alcohol, loaded with curcumin and zinc oxide, showed accelerated cell migration and proliferation during healing, as well as excellent antibacterial and anti-biofilm properties, demonstrating strong potential for the treatment of diabetic foot ulcers [50].

Nanofibers have a high load capacity, allowing them to incorporate multiple active ingredients. In recent research, a wound dressing was prepared, consisting of poly(lacturo-co-glycolide) nanofiber membranes loaded with curcumin and grafted with heparin. This design enabled the sustained release of the exogenous agent curcumin, alleviating oxidative stress and exerting anti-inflammatory effects, while capturing endogenous growth factors at the wound site, thereby promoting wound healing in diabetic rats. These dressings exhibited high tensile strength, low cytotoxicity (more than 70% viable cells), and adequate moisture control to prevent excessive wound dehydration during healing [33].

There are also reports on nanofibers composed of silk fibroin loaded with curcumin and mixed with a hydrophobic polymer (PCL) and a hydrophilic polymer (PVA) via electrospinning. These nanofibers exhibited a biphasic release pattern with rapid initial release (20–28%) during the first hour caused by degradation of the hydrophilic layer (silk and PVA), followed by sustained release of the drug molecule mediated by PCL; thus achieving a minimum effective concentration to produce the desired pharmacological response and maintaining this response for a more extended period of time. In vivo evaluation showed a maximum healing rate on day 14, with a significant decrease in wound area in a mouse model of induced diabetes [78].

### 5.3. Micelles, Nanomicelles, and Vesicles

Structures designed from amphiphilic molecules, such as micelles, nanomicelles, and vesicles, are self-assembled systems whose hydrophobic and hydrophilic segments spontaneously organize in aqueous media [80]. Vesicles exploit this organization to form internal compartments capable of transporting and storing hydrophilic drugs, as well as to functionalize their membranes with hydrophobic molecules [81]. Micelles and nanomicelles, by contrast, behave in the opposite manner, with hydrophobic molecules oriented toward the interior, thereby enabling the encapsulation of hydrophobic drugs. At the same time, the outer hydrophilic layer forms a stable crown in water, thereby facilitating drug transport and improving their solubility. These structures enable the effective encapsulation of poorly soluble molecules, such as curcumin, thereby enhancing their potential for healing and stability in the treatment of skin wounds [77].

In this way, mixed polymeric micelles loaded with curcumin were developed using chitosan, alginate, maltodextrin, nonionic pluronic-type polymeric surfactants (F127 and P123), and polysorbate 80 (Tween 80), via the thin-film hydration method. This formulation, in addition to demonstrating significant in vivo effects on wound healing, demonstrated curcumin’s antidiabetic potential, mediated by its antioxidant capacity. Curcumin was able to trap the free radicals released by bisphenol A, thereby restoring damaged β cells, lowering blood glucose, and improving the lipid profile, similar to the effects observed in the metformin-treated group [82].

Nanocellular vesicles were also studied for the treatment of refractory wounds in diabetic mice. In these systems, the drug loading rate was only 2.3%; however, favorable effects were observed on fibroblast proliferation at 40 mg/L and on the curcumin-mediated inflammatory response in macrophages. After 14 days, the wound area decreased, accompanied by extensive granulation tissue formation and collagen fiber deposition, demonstrating a synergistic effect on diabetic wound healing [49].

### 5.4. Nanoparticles

Nanoparticles are particulate structures smaller than 100 nm that have a high surface-to-volume ratio, facilitating their entry into the cellular environment and contributing to their high therapeutic efficacy and widespread use as a curcumin delivery system [4].

The manufacture of poly(lactic-co-glycolic acid) nanoparticles encapsulating curcumin has been reported, and in vivo results have shown significant improvements in wound closure rates. The NPs were shown to suppress IL-6 and TNF-α mRNA expression, proinflammatory cytokines that prolong chronic inflammation and delay healing. In turn, they modulated the JAK2/STAT signaling pathway, leading to increased expression of TGF-β, VEGF-A, and IL-10 mRNA, factors that promote cell proliferation, angiogenesis, and inflammation resolution [83].

Similarly, in another study, curcumin was encapsulated in silane hydrogel nanoparticles, and its healing and antimicrobial effects were evaluated. The nanoparticles obtained inhibited the in vitro growth of methicillin-resistant Staphylococcus aureus (MRSA) and *P. aeruginosa* in a dose-dependent manner, showing a 97.0% reduction in MRSA growth and a 59.2% reduction in *P. aeruginosa* growth. Free curcumin exhibited antimicrobial activity comparable to that of Curc-NP, reducing MRSA by approximately 95%. In vivo, Curc-NP mitigated wound expansion with a 98.1% increase from baseline, compared to 118.0% for free curcumin [84].

Antibacterial activity was also observed during the development of curcumin-loaded lignin nanoparticles, which showed in vitro efficacy against gram-positive bacteria, especially *S. aureus*. In rats, a reduction in wound area of up to 43% was observed after 12 days of treatment. In addition, increased granulation tissue formation and collagen deposition were observed, along with low matrix metalloproteinase (MMP) activity, particularly MMP9 [85]. Complementarily, in a rat model with streptozotocin-induced type I diabetes mellitus, the topical application of curcumin nanoparticles in hydrogel (Cur-NP/HG) showed a superior therapeutic effect to conventional curcumin hydrogel (Cur/HG), achieving a higher wound closure rate (93.3% vs. 58.3%), complete re-epithelialization, a significant increase in collagen deposition, and higher expression of vascular endothelial growth factor (VEGF) and aquaporin-3 (AQP3) [86].

Recent research has explored the use of gold nanoparticles (AuNPs) as advanced curcumin delivery systems, achieving sustained release and superior skin permeation relative to free curcumin. In addition, these NPs reduced minimum inhibitory concentrations and improved antibacterial activity against various gram-positive and gram-negative bacterial strains. In a diabetic wound model in rats, AuNP-loaded curcumin showed reduced oxidative stress in the wound, increased collagen deposition, intensified anti-inflammatory effects, and improved angiogenesis [87]. Another relevant study evaluated the release efficiency and therapeutic potential of chitosan NPs loaded with curcumin, achieving up to 75% release in 16 h. The effect on healing was determined in L929 murine fibroblast cells, with closures of between 45 µm for NPs compared to 59 µm for free curcumin after 24 h of treatment [88].

All these studies show that incorporating curcumin into systems such as hydrogels, nanofibers, micelles, and nanoparticles significantly enhances its therapeutic effects, achieving more efficient modulation of inflammation, enhanced antimicrobial activity, and accelerated wound healing.

## 6. Chitosan as a Healing Agent

Chitosan is a polymer obtained by deacetylation of chitin, a natural polysaccharide abundant in nature, present in the exoskeleton of crustaceans and insects, and in the mycelium of fungi [89]. It has a unique structure composed of randomly arranged β-(1,4)-D-glucosamine and N-acetyl-D-glucosamine units [90,91]. Among its many properties, biocompatibility, biodegradability, hydrophilicity, cross-linking density, mucoadhesiveness, and the ability to promote fibroblast and keratinocyte proliferation stand out, enabling controlled drug release and promoting wound healing [90,92]. The presence of amino groups in the chitosan structure also contributes to a positive zeta potential and accounts for the compound’s ease of adhesion to mucous membranes [90]. Furthermore, this compound has been shown to exert analgesic and hemostatic effects, mediated by the N-acetylglucosamine monomer, which is involved in cell proliferation, making it a promising therapeutic option for wound closure [21,93].

Chitosan also exhibits interesting antimicrobial activity due to the positive surface charge of its amino groups, which allows it to bind electrostatically to the negatively charged bacterial cell membranes, promoting structural changes in the bacterial cell that disrupt cellular organization and initiate bacterial death [94,95]. In addition, the antimicrobial effects of chitosan are attributed to destabilization of peptidoglycan, which creates an osmotic imbalance in the cell wall and disrupts the electron and oxygen transport chains, thereby affecting membrane energy stability.

Electrostatic interactions can occur both directly in gram-positive bacteria (especially pathogens such as *S. aureus*) and in gram-negative bacteria through interactions with bacterial anionic structures, with greater activity observed in the latter group [96]. These interactions tend to be long-lasting, given chitosan’s small size relative to bacteria and its low volume-to-surface-area ratio [94].

Chitosan’s physicochemical properties, such as the degree of deacetylation and molecular weight, are key structural parameters that directly affect its therapeutic properties [27,97]. In fact, molecular weight influences the solubility and antioxidant capacity of chitosan.

It has been reported that an increase in molecular weight decreases aqueous solubility due to an increase in intra- and intermolecular hydrogen bonds. Similarly, the polymer’s antioxidant capacity, attributed to the stabilization of reactive species by hydroxyl groups at the C-6 position and amino groups at the C-2 position, varies significantly between high- and low-molecular-weight chitosan (42% and 95%, respectively) [98].

The impact of this variable was also evaluated during the development of an injectable hydrogel formulation with low and high-molecular-weight chitosan (LCH and HCH), loaded with curcumin and liposomes loaded with α-tocopherol [62]. In this study, the antioxidant properties of the loaded drugs were affected by the molecular weight of chitosan. In this regard, LCH exhibited better radical scavenging (≈85%) than HCH (≈35%). It also demonstrated that molecular weight influenced the selectivity of the compound toward specific bacteria; thus, HCH formulations were more effective against *S. aureus*, while LCH formulations were more effective against *E. coli*, due to the difference in the membrane pore structure of the two pathogens [62].

On the other hand, it has been reported that increasing the degree of deacetylation (DD) enhances mechanical properties, such as tensile strength and elastic modulus. In addition, with the increase in the degree of deacetylation, the number of amino groups on carbon C2 also increases, which is directly related to antioxidant properties [99]. Taken together, these data demonstrate that high DD, low molecular weight chitosans are best suited for nanoencapsulation applications in delivery systems, as they possess higher biological activity, improved stability, and high encapsulation efficiency [100].

Structural modification is a frequently used approach for optimizing the biological properties of chitosan [95], facilitated by the large number of reactive amino groups available for cross-linking reactions [93]. On the other hand, given the high viscosity of the solutions, electrospinning of pure chitosan is not feasible; it is commonly combined with other biocompatible polymers to optimize chitosan properties [21]. Functionalization of the compound with other molecules, such as polyvinyl alcohol, is a viable option for improving the properties of chitosan [101].

Among these functionalizations is the development of a bimetallic oxide-modified chitosan structure for wound dressings. This nanohybrid scaffold exhibited good mechanical properties and excellent antimicrobial activity, thereby improving the wound closure rate [95].

In another study, an injectable hydrogel comprising chitosan-*g*-quaternized polyaniline was developed with antioxidant, antimicrobial, and coagulant properties, which improved healing in a full-thickness skin defect model evaluated in vivo. This hydrogel was electroactive and exhibited excellent tissue adhesiveness, showing greater solubility and cytocompatibility than pure quaternized chitosan [93].

It has also been shown that incorporating chitosan into other drug delivery systems enhances their intrinsic properties. For example, PLGA nanoparticles loaded with curcumin were reported, and the effect of chitosan coating on in vitro and in vivo toxicity was evaluated, yielding favorable results for the coated systems. Cell viability, assessed using an MTT (thiazolyl blue tetrazolium bromide) assay, was greater than 90% on day 1 and above 100% on days 2 and 3. In vivo toxicity studies, primarily histopathological analysis in adult Wistar rats, revealed no inflammation or other abnormalities in the evaluated tissue samples. The absence of inflammation was attributed to curcumin [90].

Thermal gelation based on the symmetrical triblock copolymer Pluronic P123 (polyethylene glycol block-polypropylene glycol block-polyethylene glycol), applied to chitosan backbones, can functionalize this compound so that it can form a gel near body temperature and become a solution below 20 °C, depending on the polymer concentration. This functional hydrogel loaded with gelatin nanofibers and curcumin exhibited synergistic effects, thereby improving its healing properties [96].

The antimicrobial and hemostatic effects, together with its ability to promote fibroblast and keratinocyte proliferation, make chitosan a potential delivery system for wound healing, particularly in infections such as those observed in UPD. These effects, mediated mainly by the amino groups of its monomeric subunit, N-acetylglucosamine, are influenced by its physicochemical properties, particularly the degree of deacetylation and molecular weight. Due to these limitations, advanced formulation of these systems is necessary, which often involves structural modification of chitosan, as well as the inclusion of other drug-carrying materials, which can also enhance the healing effects of chitosan and add advantages such as sustained and specific release, greater cell viability, and specificity of bacterial strains. Furthermore, if formulated with molecules such as curcumin, which has antibacterial, antioxidant, and anti-inflammatory properties, it may be a promising strategy for treating PDU.

## 7. Curcumin Delivery Systems with Chitosan

**Figure 9:**
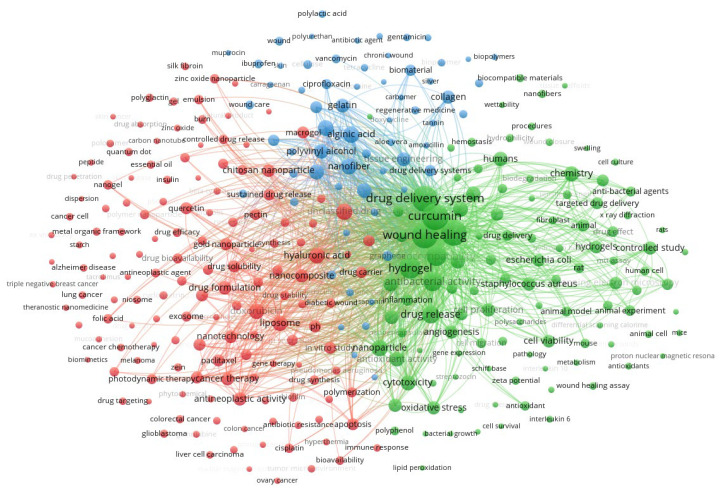
Co-occurrence network based on article weights for terms associated with the first group. The curved lines of varying thickness, representing co-occurrence, illustrate the relationships between the terms. The proximity between the elements reflects the strength of their connection, while the size of each term is determined by its frequency of occurrence: Keywords: 321. Clusters: 3. Links: 43,742. Total link strength: 227,444.

A bibliometric approach was used to identify the main research trends in the literature on chitosan-based curcumin delivery systems for wound healing. For this keyword network, terms cited at least ten times were used, yielding 321 words organized into three groups, with a total link strength of 227,444 (Figure 9). The initial search was refined by removing irrelevant terms. The red and blue clusters highlight associations between additional biomaterials in chitosan formulations and emphasize the importance of delivery systems. Meanwhile, the green cluster shows terms related to biological properties, including “angiogenesis” and “antibacterial activity.”

### 7.1. Therapeutic Properties

The administration of curcumin via chitosan nanosystems has been studied recently, with particular emphasis on manufacturing strategies and the system’s therapeutic properties [102]. Among these properties, the role of these systems in tissue repair stands out, particularly their antimicrobial, antioxidant, and re-epithelializing effects [103]. In a recent study, the impact of curcumin-chitosan nanocomplexes was evaluated in clinical isolates of non-fermenting, biofilm-producing gram-negative bacilli. This system, encapsulated by ionotropic gelation, exhibited biofilm-inhibiting activity against *Achromobacter xylosoxidans*, *Burkholderia cepacia*, and *Stenotrophomonas maltophilia*, proving to be an alternative for use in combination with trimethoprim-sulfamethoxazole [104].

In an additional study, also using the ionic gelation technique, curcumin-chitosan nanoparticles were prepared to evaluate their effect on *S. aureus* and *P. aeruginosa* biofilms. Curcumin exhibited an encapsulation efficiency of 45%, resulting in a 92% reduction in biofilms after irradiation, demonstrating its photosensitizing effects and photodynamic antibacterial activity. This activity was dose-dependent and mediated by the formation of reactive oxygen species. In addition, high viability of human fibroblasts (>85%) was observed, suggesting good biocompatibility and potential safety for topical applications [105]. In general, these antibacterial effects are more common in the formulation of chitosan–curcumin nanostructures (Cur-CS-NP) with other compounds, as demonstrated in a study in which hybrid nanocomposites based on chitosan and curcumin (CUR) were synthesized with graphene oxide (GO) and copper oxide (CuO) as antibacterial and cytotoxic drugs. This formulation, in which the Cur-CS-NP system was considered solely as an administration vehicle, demonstrated remarkable antibacterial activity against *S. aureus* and *E. coli*. In addition, it presented a large surface area, biocompatibility, high oxidative stress, and the ability to disrupt bacterial cells [60]. Likewise, the preparation of Cur-CS-NP with gold (Au) significantly improved the antimicrobial effects of curcumin on gram-positive and gram-negative bacteria under light conditions, which was superior to that obtained with free curcumin [64].

The antibacterial effects have also been evaluated in conjunction with antioxidants; in this case, in a system composed of a mixture of three curcuminoids (curcumin, demethoxycurcumin, and bisdemethoxycurcumin), termed curcumin-C3. The encapsulation of this compound in chitosan nanoparticles via ionic gelation showed high efficiency (>90%), excellent drug release, strong antioxidant potential, and notable inhibition of *E. coli* and *S. aureus*, demonstrating that chitosan is a suitable carrier for curcumin-C3 nanoparticles [58].

These antioxidant properties were also demonstrated in rats poisoned with phenpropathine (FNE) using chitosan nanoparticles loaded with curcumin, prepared by ionic gelation. This treatment significantly decreased ROS and MDA levels and increased SOD, CAT, and GSH contents, thereby alleviating oxidative stress, lipid peroxidation, and mitochondrial dysfunction. In turn, a protective effect of curcumin was evidenced, mediated by the negative regulation of Bax and Caspase-3 gene expression during the joint administration of Cur-CS-NP + FNE, underscoring the therapeutic potential of this system to prevent and mitigate oxidative damage [51].

Similarly, the antioxidant capacity of a system composed of chitosan-coated solid lipid nanoparticles loaded with curcumin (CuCsSLN) was demonstrated, which provided sustained delivery for up to 24 h and achieved a reduction in cytotoxic effects, ROS formation, mitochondrial membrane collapse, lipid peroxidation, and oxidative stress caused by the use of celecoxib (20 µg/mL), in addition to a marked cytoprotective effect. These results were superior to those obtained with free curcumin, highlighting the importance of the chitosan delivery system in enhancing curcumin’s bioavailability and antioxidant effects [106].

The antioxidant properties of chitosan–curcumin nanostructures (Cur-CS-NP) are of particular interest for pathologies characterized by high oxidative stress, such as diabetic nephropathy. A recent study found decreased caspase-3 activity, MDA, and ROS levels, accompanied by increased SOD levels. On the other hand, inflammatory factors such as TGF-β1, TNF-α, and IL-6 were reduced, while IL-10 and podocin secretion increased. In this way, oxidative stress and inflammation were reduced, thereby decreasing apoptosis and improving the development of diabetic nephropathy [107].

### 7.2. Applications for Wound Healing

Curcumin loading in chitosan systems has demonstrated isolated antimicrobial and antioxidant effects, which, together, could accelerate wound healing. In recent years, the role of curcumin-loaded CS nanoparticles in promoting wound healing has been extensively studied [102].

Recently, the application of chitosan/polyvinyl alcohol/hydroxyapatite nanofibers loaded with curcumin as wound dressings with enhanced antimicrobial activity was investigated. These electrospun nanofibers demonstrated significant antimicrobial activity against *E. coli* and *S. aureus*, with inhibition diameters of 11.6 mm and 14.3 mm, respectively. Free curcumin, on the other hand, showed no zones of microbial inhibition, highlighting this system as a promising candidate for applications in infected wound dressings [101]. A series of chitosan scaffolds loaded with curcumin/TiO_2_ complexes (CUT) was also fabricated to evaluate their healing capacity in MRSA-infected wounds in vivo and their in vitro antimicrobial activity. Curcumin powder showed an inhibition zone of 1.5 mm against *E. coli* and 2.0 mm for *S. aureus*. In contrast, CUT scaffolds exhibited larger inhibition zones, ranging from 4.8 to 8.1 mm. In terms of wound contraction, curcumin recorded a value of 60.58% on day 14, while the scaffolds showed a gradual increase, reaching values of up to 78.47% [108].

Electrospun curcumin-chitosan nanofibers represent a promising strategy for improving curcumin’s bioavailability, tissue retention, and transdermal delivery. One study, in which poly (ε-caprolactone) nanofibers with chitosan and curcumin were successfully prepared, demonstrated high antibacterial and antioxidant performance accompanied by improvements in the proliferation rate of human dermal fibroblasts, achieving a healing rate of 98.5% in uninfected wounds and 96.4% in wounds infected with MRSA after 15 days of treatment. This system stands out for its antimicrobial activity and its role in wound healing [21].

Other unique properties of these systems, advantageous for wound healing, were explored in the design of nanofibers composed of grafted chitosan and polypropylene carbonate to encapsulate curcumin, and in the manufacture of electrospun nanofibers free of polyvinyl alcohol. The nanofibers obtained were evaluated in vivo, showing excellent antioxidant capacity, high collagen deposition, and a wound closure rate of approximately 100% on day 21 of treatment, using a curcumin load of only 10%, thanks to the ultra-fine diameter, high volume ratio, and three-dimensional structure of the nanofibers [109].

In addition to electrospun nanofibers, nanoparticles have been studied for their role in wound healing. In this context, the effects of curcumin-polyethylene glycol encapsulated in chitosan-gelatin nanoparticles (C-PEG-CGNP) on burn wound healing in rats were evaluated. The results showed that C-PEG-CGNP significantly reduced the inflammatory phase and increased fibroblast distribution in the wound area, facilitating rapid epithelialization. These findings suggest a potential advantage for improving cell proliferation and burn-wound healing, highlighting the anti-inflammatory properties of this system [110].

Interventions aimed at treating various symptoms associated with wound healing, such as chronic inflammation, excessive oxidative stress, and microbial proliferation, represent a comprehensive approach and are preferable to formulations that address the re-epithelialization process in a single-factor manner [2]. In this regard, the recent development of bilayer skin patches with antibacterial, antioxidant, and anti-inflammatory properties is an interesting alternative. These patches, composed of an upper layer of polycaprolactone (PCL) and chitosan (CS), and a bottom layer of polyvinyl alcohol (PVA) with curcumin nanoparticles and soluble eggshell membrane protein (SESM), showed a reduction in the production of ROS, cytokines, and inflammatory markers in mouse T cells mediated by curcumin and an increase in the migration of adult human dermal fibroblasts, as contributed by SESM. In addition, they exhibited excellent hemocompatibility and antibacterial properties, provided adequate support for cell recruitment and extracellular matrix deposition, and demonstrated good absorption of wound exudates, thereby enabling optimal healing in a full-thickness excision wound model in rats [23]. However, the aforementioned studies include an additional component in the chitosan–curcumin system, thereby preventing the evaluation of the efficacy of the system of interest alone in wound healing. To address this limitation, recent research prepared chitosan/curcumin (CS/Cur) nanoparticles without any additional compounds and evaluated their anti-inflammatory, antioxidant, and antibacterial properties to assess their potential for wound healing. The results indicated that the system was effective as an anti-inflammatory, exhibited high antibacterial activity, and had antioxidant activity of 41.6%. In addition, cell growth in the affected tissue increased by up to 616% over 7 days, reinforcing its healing characteristics [111].

### 7.3. Application of the Curcumin-Chitosan System in Diabetic Foot Ulcer Healing

The healing of diabetic foot ulcers is a significant challenge due to the multifactorial nature of the healing process and its associated complications. These complications have highlighted the need to design multifunctional dressings capable of providing different properties to optimize the particularly slow healing process of this type of lesion [112].

In a recent study, multifunctional electrospun nanofibers composed of chitosan-polyvinyl alcohol, loaded with curcumin and zinc oxide, were developed and evaluated for accelerating the healing of diabetic wounds. For this purpose, diabetes was induced in adult Wistar rats by administering streptozotocin, a compound that selectively destroys pancreatic islet β cells, thereby generating an experimental model of moderate diabetes. The results of the in vitro tests showed increased cell migration at the wound site in the immortalized human keratinocyte cell line (HaCaT), a significant improvement in wound-contracting capacity over 14 days, and histological studies demonstrated that nanofibers formed collagen during healing. In addition, antibacterial efficacy against *S. aureus* and *P. aeruginosa* was confirmed, thereby enabling the manufactured scaffolds to prevent infections in diabetic wounds. The mechanical strength, biodegradability, and water retention capacity of these structures, which promote healing in DFU, are also noteworthy [50].

Similarly, an in vivo study in Sprague–Dawley rats as a biomodels confirmed that a high concentration of chitosan, in combination with curcumin, significantly improves healing of diabetic wounds. To develop this study, formulations with varying chitosan-to–methylcellulose ratios were prepared to establish an appropriate delivery system for curcumin. The results showed that the scaffold prepared with a 3:1 ratio (chitosan: methylcellulose) exhibited intense bactericidal activity against *E. coli* and *S. aureus*, as well as erythrocyte interaction, facilitated by chitosan’s blood affinity, resulting in a coagulation time of ~32 ± 2 s and rapid hemostasis. Histopathological analyses of punch biopsies performed on days 0, 7, and 14 showed that curcumin induced symmetrical granulation tissue, characterized by fibroblast proliferation, increased collagen deposition, and rapid re-epithelialization, resulting in wound closure in diabetic animals by day 14. Other properties, such as a microporous structure and biodegradability, together with the aforementioned effects, confer on these biocomposite scaffolds great potential to promote faster hemostatic action and diabetic wound healing [113].

Finally, chitosan nanoparticles loaded with curcumin (Cur-CS-NP) were developed, and their potential for diabetic wound healing was investigated. These nanoparticles induced a significantly smaller decrease in the expression of inflammatory factors, including TLR-4, NF-κB, TNF-α, and IL-6, than the free curcumin group (*p* < 0.05), thereby accelerating healing in a streptozotocin-induced diabetic rat model. This was evidenced by the wound closure rates reported by the authors: while free curcumin had a rate of 79.7% on day 7 of treatment, Cur-CS-NPs induced higher closure rates, reaching 90.6% on day 7. Therefore, Cur-CS-NPs represent an efficient strategy for accelerating diabetic wound healing by reducing inflammation [11]. Table 1 summarizes recent advances in research on the healing properties of chitosan–curcumin nanoformulations.

## 8. Discussion

The objective of this review was to comprehensively examine the current literature on the mechanisms underlying the healing properties of chitosan–curcumin nanostructures in the treatment of DFU and to observe how this system enhances the therapeutic effects of free curcumin.

According to the findings, topical administration of Cur-CS-NP shows marked anti-inflammatory, antimicrobial, antioxidant, and re-epithelializing effects. The anti-inflammatory effects are primarily mediated by the polarization of M1 macrophages toward M2 macrophages and by the inhibition of inflammatory mediators, including COX-2, NF-κB, TNF-α, and IL-6 [2,43,114]. Both effects are enhanced by the sustained release of curcumin from its delivery system, thereby reducing inflammation throughout the healing process and supporting DFU regeneration.

On the other hand, the prolongation of the inflammatory phase is attributed, among other factors, to the excess of superoxide anions present in the DFU [28]. The presence of hydroxyl and phenolic groups in the structure of curcumin provides it with direct antioxidant capacity through the elimination of ROS and indirect antioxidant capacity by increasing the activity of antioxidant enzymes such as SOD, CAT, and GSH [23,107]. In addition, it exerts a significant cytoprotective effect on proliferative fibroblasts, which promotes tissue regeneration. The sustained release of curcumin from chitosan delivery systems prolongs its circulation time and its interaction with ROS, thereby increasing its potential antioxidant effects.

Regarding the mechanisms underlying the antimicrobial effects of Cur-CS-NP, it is important to highlight both the individual effects of its components and the properties arising from their combined formulation. On the one hand, curcumin can enter bacterial cells via its lipophilic properties and triggers biochemical changes that lead to the filtration of cellular contents and, ultimately, to lysis; on the other hand, it inhibits cell division due to its chemical structure. Chitosan, on the other hand, can bind electrostatically to the negatively charged surfaces of bacterial membranes via its positively charged amino groups, thereby disrupting cellular organization and causing cell death. The combination of these components can confer selectivity in inhibition, targeting specific strains. It also overcomes the limitations associated with low bioavailability and prolongs the antibacterial effects through sustained release in acidic microenvironments, present in bacterial infections [43,56].

These systems have also been shown to have significant re-epithelialization capacity, characterized by high collagen deposition, increased fibroblast viability, and hemostatic properties [21,93]. These properties are directly related to the microporous structure of Cur-CS-NPs, their water-retention capacity, and their exudate-absorbing capacity at the wound site, which promote healing in DFU. However, it is crucial to consider key factors such as the loading capacity and the technique to be used to obtain the nanosystems, with particular emphasis on the electrospinning technique, widely used due to its high loading capacity, as well as the mechanical and porous properties it confers on the scaffolds, giving them remarkable strength. This technique also enables the production of nanofibers that simulate the structure of the natural extracellular matrix [50,109].

The anti-inflammatory and antioxidant properties of the Cur-CS-NP system are inherent to curcumin. The incorporation of chitosan as an encapsulation system primarily improves bioavailability, facilitates drug administration, prolongs its residence time at the site of action, and maintains sustained therapeutic levels. This aspect is particularly relevant in the context of DFUs, given that an environment characterized by high oxidative stress and chronic inflammation requires a sustained supply of the active ingredient to promote healing. However, chitosan does not enhance curcumin’s anti-inflammatory or antioxidant properties. The antimicrobial and re-epithelializing effects, by contrast, cannot be explained solely by improved bioavailability; rather, they arise from the combined properties of curcumin and chitosan. In contrast, curcumin alters the bacterial membrane and inhibits cell division [55,60], and chitosan enhances antibacterial activity through its cationic charge and contributes to hemostasis and cell proliferation [94,95]. Thus, the combination of both components not only amplifies the individual effects but also integrates anti-inflammatory, antioxidant, antibacterial, and re-epithelializing activities into a single system, making it a comprehensive therapeutic alternative to the limitations of free curcumin. This evidence suggests that the Cur-CS-NP system should not be considered solely as a delivery vehicle, but as a co-therapy model in which the carrier provides bioactive properties that enhance clinical outcomes in the treatment of DFU.

In all cases, these effects exceed those observed with free curcumin, highlighting the need to incorporate this compound into nanostructures to improve its absorption and bioavailability, thereby enabling its therapeutic effects to be exploited.

Finally, barriers to transitioning these systems to clinical application have been identified: industrial scalability, prolonged safety studies, dosing, and batch-to-batch reproducibility. Addressing these limitations, along with the lack of standardization of characterization and design protocols for these systems, would be a key step toward future clinical application in patients with DFU.

## 9. Future Prospects, Challenges, and Limitations in the Medical Application of Chitosan-Nanoencapsulated Curcumin

Despite the favorable results of the studies analyzed, the evidence remains heterogeneous. There is high variability in the study parameters, such as the doses used, the physicochemical characteristics of the systems (loading rate and release percentage), as well as in the cell lines and animal models used, which makes it difficult to compare the results directly and highlights the need to move towards more uniform experimental designs.

The literature provides limited evidence of the simultaneous anti-inflammatory, antioxidant, and antimicrobial effects of nanoencapsulated curcumin, using chitosan as the sole carrier material. Even fewer studies have applied these systems to the treatment of DFU. Generally, one or two properties are investigated separately. When all are evaluated, they are often combined with other compounds to form nanostructures or evaluated for applications beyond wound healing, making it even more difficult to identify research focused on specific DFU applications.

Another challenge in developing these systems is reproducibility between batches. It is essential to identify and control process parameters whose variability can directly affect the quality and application of these formulations. Despite the widespread dissemination of satisfactory therapeutic results, many studies provide limited information or lack robust statistical analysis. Therefore, reproducibility in the manufacturing process is a limiting factor for the scalability of these systems and even more so for their clinical application.

Among these critical parameters are the molecular weight of chitosan and the degree of deacetylation. Careful selection of these parameters is necessary to ensure adequate delivery of curcumin and obtain the expected biological activities. However, the lack of uniformity in the variables considered across the studies reviewed, together with the frequent incorporation of other biomaterials into nanoformulation designs, makes direct comparison difficult. Consequently, the impact of chitosan’s physicochemical variability on curcumin release and therapeutic outcomes remains inconclusive.

Likewise, to maximize the positive impact of these systems, it is important to highlight the need for future research that addresses Chitosan’s ability to encapsulate curcumin without being associated with other molecules and that jointly evaluates its anti-inflammatory, antioxidant, and antimicrobial properties, specifically for the healing of UPD. It would also be important to elucidate the effects at the cellular and molecular levels and to conduct clinical trials to validate the efficacy of the formulations.

It is essential to thoroughly investigate the dose–response relationships of these systems to optimize efficacy and minimize cytotoxicity, and to conduct long-term safety studies and standardize design and characterization processes to select the most suitable systems for use in patients with DPU.

Finally, it would be interesting to explore the local antidiabetic capacity of curcumin to further enhance diabetic wound-healing efficacy.

## 10. Conclusions

Significant efforts have been made to develop various types of chitosan nanostructures loaded with curcumin to expand curcumin’s therapeutic applications in the healing of complex wounds, such as diabetic foot ulcers. This literature review revealed the mechanisms underlying the anti-inflammatory, antioxidant, and antimicrobial effects of these nanostructures, which are directly related to the chemical structures of both molecules. These effects are enhanced by the controlled release and improved bioavailability provided by these systems, ensuring an effective dose over prolonged periods. A relevant finding indicates that chitosan not only provides a more efficient delivery system for curcumin therapy but also confers additional antimicrobial and hemostatic effects that promote closure of complex wounds. Together, these systems demonstrate great therapeutic potential for treating diabetic foot ulcers, combining high loading efficiency, reduced healing time, and smart-release strategies. Therefore, DFU treatment with chitosan and curcumin nanostructures is considered promising and shows multiple therapeutic advantages over free curcumin.

## Figures and Tables

**Table 1:** Healing properties of DFU associated with recent chitosan–curcumin nanoformulations.

Type of Formulation	Associated Components	Manufacturing Techniques	Experimental Model	Pathological Model	Comparative Curcumin Group	Properties	Reference
Nanofibers	Chitosan Curcumin Polyvinyl Alcohol Hydroxyapatite	Electrospinning	In vitro	Non-diabetic wound	Yes	Antimicrobial Fibroblast viability	[101]
Nanofibers	Poly(ε-caprolactone) Chitosan Curcumin	Electrospinning	In vitro/in vivo	Non-diabetic wound (Infected)	No	Antimicrobial Fibroblast proliferation Antioxidant	[21]
Nanofibers	Polypropylene polycarbonate Chitosan Curcumin	Electrospinning	In vitro/in vivo	Non-diabetic wound	No	Antioxidant Collagen deposition	[109]
Nanoparticles	Polyethylene glycol Gelatin Chitosan Curcumin	Ionic gelation	In vivo	Non-diabetic wound (burn)	No	Anti-inflammatory Fibroblast viability	[110]
Biphasic dermal patches	Polycaprolactone Chitosan Polyvinyl alcohol Curcumin Soluble egg shell membrane protein (SESM)	Solvent precipitation assisted by sonication	In vitro/in vivo	Non-diabetic wound	No	Antioxidant Anti-inflammatory Fibroblast proliferation Hemocompatibility Antimicrobial Cell recruitment Extracellular matrix deposition	[23]
Nanofibers	Chitosan Polyvinyl alcohol Curcumin Zinc oxide	Electrospinning	In vitro/in vivo	Diabetic wound	No	Antimicrobial Collagen deposition Cell migration	[50]
Three-dimensional biocomposite scaffold	Chitosan Methylcellulose Curcumin	Lyophilization	In vitro/in vivo	Diabetic wound	Yes	Antimicrobial Hemostatic Fibroblast proliferation Collagen deposition	[113]
Nanoparticles	Chitosan Curcumin	Ionic crosslinking	In vitro/in vivo	Diabetic wound	Yes	Anti-inflammatory Cell migration Collagen deposition Fibroblast proliferation	[11]

## Data Availability

The original contributions presented in this study are included in the article/Supplementary Materials. Further inquiries can be directed to the corresponding author.

## Supplementary Materials

The following supporting information can be downloaded at: https://www.mdpi.com/article/10.3390/polym18040511/s1, Scheme S1. Flowchart of the search strategy based on the PRISMA methodology, 2020 [115].
