# Supplementary material for: Chitosan-Nanoencapsulated Curcumin for the Treatment of Diabetic Foot Ulcers: A Review
Source: Polymers (Basel). 2026 Feb 19;18(4):511. doi: 10.3390/polym18040511 (PMC12944176; doi:10.3390/polym18040511)
Supplement: Supplementary file 1 [file polymers-18-00511-s001.zip › polymers-4130986-supplementary.pdf]

Review

Chitosan-Nanoencapsulated Curcumin for the Treatment of Diabetic Foot Ulcers: A Review

Laura Andrea Gómez-de la Cruz [1], Juan David Rodríguez Macías [2] and Carlos David Grande-Tovar [1,*]

[1] Grupo de Investigación de Fotoquímica y Fotobiología, Programa de Química, Universidad del Atlántico, Carrera 30 No. 8–49, Puerto Colombia 081007, Colombia; lauragomezdlc@hotmail.com
[2] Programa de Medicina, Facultad de Ciencias de la Salud, Exactas y Naturales, Universidad Libre, Km 5 Vía Puerto Colombia, Barranquilla 081007, Colombia; juand.rodriguezm@unilibre.edu.co
* Correspondence: carlosgrande@mail.uniatlantico.edu.co

Supplementary Material

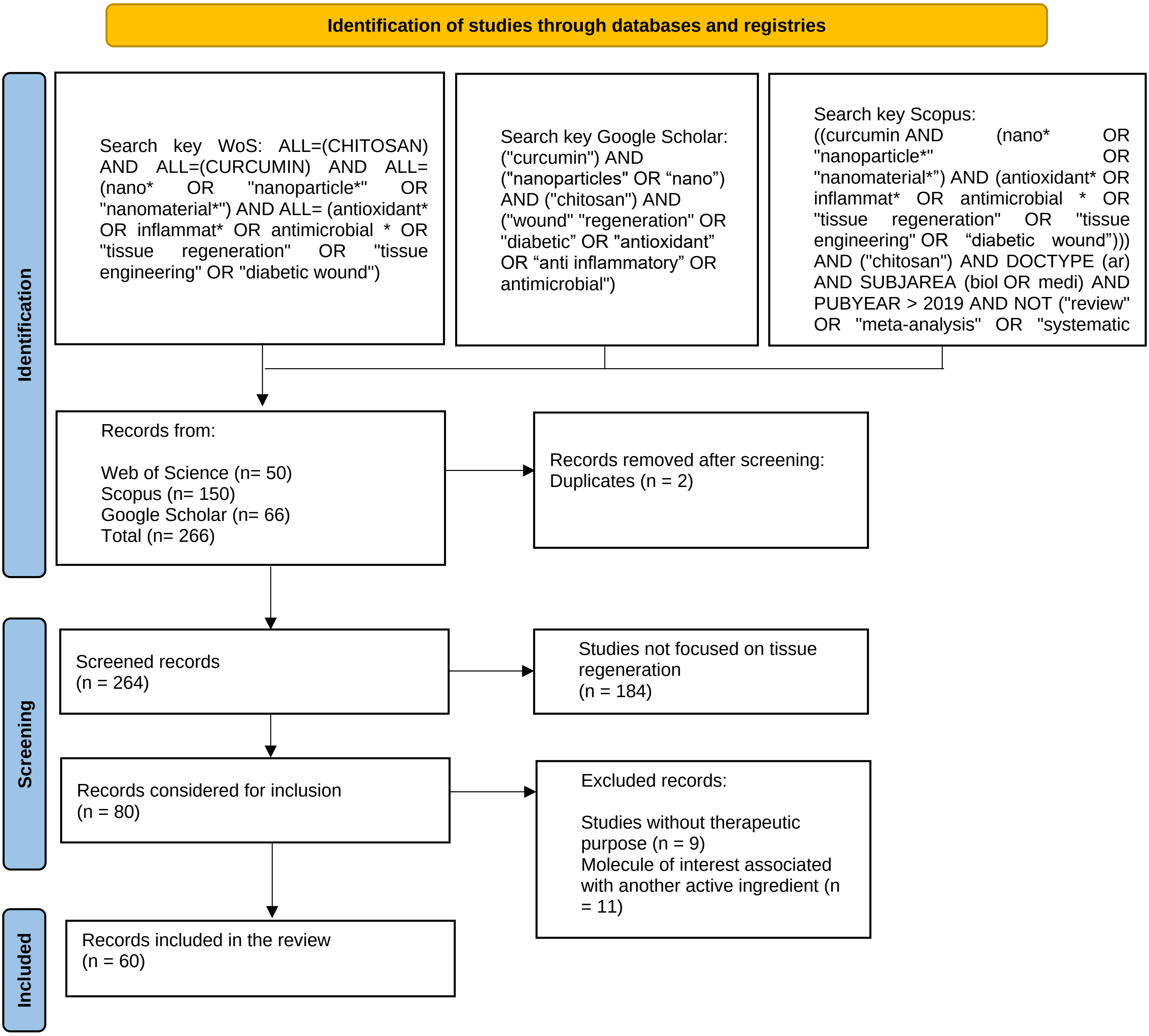

Scheme S1. Flowchart of the search strategy based on the PRISMA methodology, 2020.

Source: Page MJ, et al. BMJ 2021;372:n71. doi: 10.1136/bmj.n71.

This work is licensed under CC BY 4.0. To view a copy of this license, visit https://creativecommons.org/licenses/by/4.0/
